# A Retrospective Analysis of Epidemiological Factors and Surgical Outcomes of Patients With Ocular Trauma at Hospital Kuala Lumpur, Malaysia

**DOI:** 10.7759/cureus.75941

**Published:** 2024-12-18

**Authors:** Chua Ter Wei, Jamalia Rahmat, Rosilah Mohamad, Safinaz Mohd Khialdin

**Affiliations:** 1 Department of Ophthalmology, Hospital Kuala Lumpur, Kuala Lumpur, MYS; 2 Department of Ophthalmology, Hospital Universiti Kebangsaan Malaysia, Kuala Lumpur, MYS

**Keywords:** emergency ocular surgery, epidemiology of ocular trauma, etiology of ocular trauma, ocular trauma, ocular trauma score (ots), traumatic blindness

## Abstract

Background

The objective of this review is to study the demographics, aetiology, clinical findings, and surgical outcomes of patients who presented with ocular trauma and underwent emergency operations at Hospital Kuala Lumpur, Malaysia.

Methods

Patients who presented from 2016 to 2023 with ocular trauma and underwent emergency ocular surgery were identified and their medical records were reviewed with respect to demographics, mechanism of injury, type of injury, initial presenting visual acuity and final visual acuity after surgery. Components of the ocular trauma score (OTS) were also recorded.

Results

A total of 218 eyes from 214 patients were analysed. The majority of patients are male (185, 86.4%) and are young adults from the age of 21-40 (105, 49.1%). Industrial or workplace injuries are the most common cause of ocular injury (77, 36.5%) followed by domestic household accidents (43, 20.4%) and motor vehicular accidents (40, 19.0%). A total of 123 (56.4%) had open-globe injuries, 63 (28.9%) had injuries to their adnexa and 32 (14.7%) had a closed-globe injury. The majority of patients (61, 96.8%) who sustained adnexal injuries presented with mild visual impairment and had a high OTS. An overwhelming majority (96.4%) had a final visual acuity of at least 20/40 or better. For patients with closed-globe injuries, slightly more than half of them (18, 56.2%) had an OTS of 5, eight (25%) had an OTS of 4 and six (18.8%) had an OTS of 3. After surgical intervention for closed-globe injuries, the majority of patients with OTS 5 (15, 83.3%) and OTS 4 (6, 75%) had a final visual acuity of 20/40 or better. Four (66.7%) of patients with OTS 3 had a final visual acuity of 1/200 to 19/200. Most patients with open-globe injury presented with severe visual impairment and had a low OTS, which carries a poorer prognosis. Forty-one (33.3%) had OTS 1, 19 (15.4%) had OTS 2, 31 (25.2.%) had OTS 3, 31 (25.2%) had OTS 4 and only six (5.4%) had OTS 5. Even after surgical intervention to repair these open-globe injuries, 56.1% (23) of patients with OTS 1 had a vision of no perception to light (NPL) and 26.8% (11) had severe visual impairment of perception to light (PL) or hand movements (HM) after surgery. Thirty-three per cent (five) of patients with open-globe injuries and OTS 4 had final visual acuity of only PL or HM. The percentage of a better final visual outcome gradually increased for patients with higher OTS scores.

Conclusion

We have identified that young male adults are most at risk for sustaining ocular trauma while at the workplace and are more likely to sustain open-globe injury with lasting visual impairment. Our study also found that the OTS is a helpful tool to help healthcare practitioners predict the final visual acuity based on initial findings. Targeted approaches should be taken to reduce visual morbidity.

## Introduction

Ocular injuries continue to be a significant problem worldwide. It is a common cause of ophthalmology consultation and often results in a diminished quality of life by loss of visual acuity. This will lead to major financial implications for individuals, the healthcare system and also communities. Numerous studies have identified numerous risk factors that are common in people who sustain ocular injuries such as young adults, male gender and industries or construction workers.

Multiple characteristics have also been identified to help predict the visual outcome of an ocular injury. The Ocular Trauma Score (OTS) has been used by ophthalmologists to assess the severity of ocular trauma and predict the visual prognosis of patients based on certain characteristics of the injury. It helps clinicians evaluate the likelihood of a patient achieving various levels of visual acuity after treatment. This can assist in decision-making, patient counselling, and resource allocation in emergency and trauma settings.

## Materials and methods

Objectives

This eight-year retrospective study aims to identify the demographics, aetiology, clinical findings, and surgical outcomes of patients who underwent emergency operations for ocular trauma at Hospital Kuala Lumpur, Malaysia.

Methodology

This is a retrospective descriptive study that targeted all the patients who underwent emergency surgery for ocular trauma at Hospital Kuala Lumpur over an eight-year period from January 2016 to December 2023. Cases were identified from operation theatre surgical records and their medical records were traced and reviewed.

The following data were collected from the medical records: Demographic data such as age, gender, ethnicity and nationality; date of surgery; type of surgery; mechanism of injury; initial presenting visual acuity; final visual acuity; whether the injury was open-globe or closed-globe; involvement of orbital adnexa. Components of the OTS were also recorded. These include the presence of globe rupture, the presence of endophthalmitis, perforating injury, the presence of retinal detachment and the presence of a relative afferent pupillary defect (RAPD) [1].

The definition of the terms is according to the Birmingham Eye Trauma Terminology. A closed-globe injury indicates no full-thickness injury while an open-globe injury indicates a full-thickness injury. Globe rupture is a full-thickness injury caused by a blunt object while lacerations are caused by a sharp object. Penetrating injuries have a single entry wound with no exit wounds, if there is the presence of more than one entrance wound then it must be from different agents. Perforating injuries are full-thickness wounds with an entrance and exit injury [2].

Table 1 shows the method of calculation for the OTS. The initial vision will give us the starting points and the presence of variables will deduct points from the initial points. The final points can be used to categorize the patient into different OTS grades and the likelihood of the final visual acuity can be predicted as seen in Table 2 [1].

**Table 1:** Ocular Trauma Score Calculation Reference: [1] NPL: No perception to light; PL: Perception to light; HM: Hand movement

Variable	Raw Points
Initial Vision	
NPL =	60
PL or HM=	70
1/200 to 19/200	80
20/200 to 20/50	90
> 20/40	100
Globe Rupture	-23
Endophthalmitis	-17
Perforating Injury	-14
Retinal Detachment	-11
Relative Afferent Pupillary Defect (RAPD)	-10
Ocular Trauma Score = Sum or Raw Points	

**Table 2:** Ocular Trauma Score and Estimated Probability of Follow-Up Visual Acuity Category at Six Months Reference: [1] OTS: Ocular Trauma Score; NPL: No perception to light; PL: Perception to light; HM: Hand movement

Raw Points Sum	OTS	NPL	PL/HM	1/200 to 19/200	20/200 to 20/50	> 20/40
0-44	1	73%	17%	7%	2%	1%
45-65	2	28%	26%	18%	13%	15%
66-80	3	2%	11%	15%	28%	44%
81-91	4	1%	2%	2%	21%	74%
92-100	5	0%	1%	2%	5%	92%

The inclusion criteria consisted of all patients who underwent emergency operations for ocular trauma at Hospital Kuala Lumpur from 2016 to 2023. The exclusion criteria included patients with missing or incomplete medical records and those who were transferred to other hospitals for subsequent eye operations.

## Results

A total of 218 eyes from 214 patients were included in this review. The demographic data is tabulated in the following tables. Table 3 shows the gender of the patients included in the study. The majority of patients are male (n=185, 86.4%) while female patients constitute 13.6% (n=29).

**Table 3:** Gender

Gender	Number	%
Male	185	86.4
Female	29	13.6

Table 4 shows the age of patients included in this study. The majority of patients are young adults from the age of 21 to 40 (n=105, 49.1%). The mean age of all patients is 32.7 ± 2.5 with a range of 1 to 86 years old. The mean age of males was 34.7 ± 2.7 with a range of 1 to 86. The mean age of females was 22.1 ± 5.6 with a range of 1 to 62 years old.

**Table 4:** Age

Age	Number	%
1-10	25	11.7
11-20	24	11.2
21-30	62	29.0
31-40	43	20.1
41-50	28	13.1
51-60	11	5.1
>61	21	9.8

Table 5 shows the ethnicity of patients included in this study. The majority of patients (71.5%, n=153) are Malaysians. Among the Malaysian patients, 44.9% (n=96) were Malays, 13.5% (n=29) were Indians, 11.7% (n=25) were Chinese, and the remaining 1.5% (n=3) were of other ethnic groups.

**Table 5:** Race and Ethnicity

Ethnicity	Number	%
Malay	96	44.9
Indian	29	13.5
Chinese	25	11.7
Iban	1	0.5
Sabahan	2	1
Non-Malaysians	61	28.5
- Bangladeshi	21	
- Burmese	13	
- Chinese National	1	
- Indian National	8	
- Indonesian	9	
- Iranian	1	
- Nepalese	1	
- Pakistani	3	
- Filipino	2	
- Sri Lankan	1	
- Thai	1	

Table 6 shows the laterality of eye injuries of patients included in this study. A total of 104 patients had injuries to their right eye, while 106 patients had injuries to their left eye. Four patients had bilateral eye injuries.

**Table 6:** Laterality

Laterality	Number
Right Eye	104
Left Eye	106
Both Eyes	4

Table 7 shows the aetiology of injuries of patients included in this study. Industrial or workplace injuries are the most common cause of ocular injury (n=77, 36.5%) followed by domestic household accidents (n=43, 20.4%) and motor vehicular accidents (n=40, 19.0%). For workplace-related injuries, 31.2% (n=24) were caused by metal pieces, 23.4% (n=18) by nails, 14.3% (n=11) by stones, 14.3% (n=11) by wires and 10.4% (n=8) by glass.

**Table 7:** Aetiology of Injury MVA: Motor vehicular accident

Aetiology	N	%
Workplace/Industrial	77	36.5
Domestic Accident	43	20.4
MVA	40	19.0
Fall	26	12.3
Assault	14	6.6
Animal	8	3.8
Sports	2	1
Firecracker	1	0.5

Table 8 shows the objects that caused workplace or industrial accidents. The most common objects are metal objects (n=24, 31.2%) followed by nails (n=18, 23.4%), stones (n=11, 14.3%), wires (n=18, 14.3%), glass (n=8, 10.4%) and plastic pipes (n=5, 6.5%).

**Table 8:** Workplace and Industrial Accidents Objects

Workplace/Industrial Accidents Objects	N	%
Metal	24	31.2
Nail	18	23.4
Stone	11	14.3
Wire	11	14.3
Glass	8	10.4
Plastic Pipe	5	6.5

Table 9 shows the type of injuries of patients included in this study. A total of 56.4% (n=123) had open-globe injury, 28.9% (n=63) had injury to their adnexa while 14.7% (n=32) had a closed-globe injury.

**Table 9:** Type of Injury

Injury Type	N	%
Adnexa	63	28.9
Open	123	56.4
Closed	32	14.7

Table 10 shows the OTS and visual outcomes of patients who sustained adnexal injuries. The majority of patients (61 of 63, 96.8%) who sustained adnexal injuries presented with mild visual impairment and had a high OTS. A high percentage of these patients had a final visual acuity of 20/40 or better.

**Table 10:** Ocular Trauma Score and Visual Outcome of Adnexal Injuries After Surgery OTS: Ocular Trauma Score; NPL: No perception to light; PL: Perception to light; HM: Hand movement; N: Number

OTS	NPL		PL/HM		1/200 to 19/200		20/200 to 20/50		>20/40		N
1	0	0%	0	0%	0	0%	0	0%	0	0%	0
2	0	0%	0	0%	0	0%	0	0%	0	0%	0
3	0	0%	0	0%	0	0%	0	0%	1	100%	1
4	0	0%	0	0%	0	0%	0	0%	1	100%	1
5	0	0%	0	0%	0	0%	4	6.6%	57	93.4%	61

Table 11 shows the OTS of patients who sustained closed-globe Injuries. Slightly more than half of them (18 of 32, 56.2%) had an OTS of 5, eight (25%) had an OTS of 4 and six (18.8%) had an OTS of 3.

**Table 11:** Ocular Trauma Score (OTS) of Closed-Globe Injuries

OTS	Number	%
1	0	0
2	0	0
3	6	18.8
4	8	25
5	18	56.2

Table 12 shows the visual outcome of patients who underwent surgery for closed-globe injuries. After surgical intervention for closed-globe injuries, the majority of patients with an OTS of 5 (n=15, 83.3%) and an OTS of 4 (n=6, 75%) had a final visual acuity of 20/40 or better. Four (80%) of patients with an OTS of 3 had a final visual acuity of 1/200 to 19/200.

**Table 12:** Ocular Trauma Score and Visual Outcome of Closed-Globe Injuries After Surgery OTS: Ocular Trauma Score; NPL: No perception to light; PL: Perception to light; HM: Hand movement; N: Number

OTS	NPL		PL/HM		1/200 to 19/200		20/200 to 20/50		>20/40		N
1	0	0%	0	0%	0	0%	0	0%	0	0%	0
2	0	0%	0	0%	0	0%	0	0%	0	0%	0
3	0	0%	0	0%	4	66.7%	2	33.3%	0	0%	6
4	0	0%	0	0%	0	0%	2	25%	6	75%	8
5	0	0%	0	0%	0	0%	3	16.7%	15	83.3%	18

Table 13 shows the OTS of patients who sustained open-globe Injuries. Most patients with open-globe injury presented with severe visual impairment and had a low OTS, which carries a poorer prognosis. Forty-one (33.3%) had an OTS of 1, 19 (15.4%) had an OTS of 2, 31 (25.2%) had an OTS of 3, 26 (21.7%) had an OTS of 4 and only six (4.9%) had an OTS of 5.

**Table 13:** Ocular Trauma Score (OTS) of Open-Globe Injuries

OTS	Number	%
1	41	33.3
2	19	15.4
3	31	25.2
4	26	21.7
5	6	4.9

Table 14 shows the visual outcome of patients who underwent surgery for open-globe injuries. Even after surgical intervention to repair these open-globe injuries, 56.1% (n=23) of patients with an OTS of 1 had a vision of no perception to light (NPL) and 26.8% (n=11) had severe visual impairment of perception to light (PL) or hand movement (HM) after surgery. For patients with open-globe injuries and an OTS of 2, 15.8% (n=3) of them had a final visual acuity of NPL and 31.6% (n=6) had a final visual acuity of only PL or HM. The percentage of a better final visual outcome gradually increased for patients with higher OTS scores.

**Table 14:** Ocular Trauma Score and Visual Outcome of Open-Globe Injuries After Surgery OTS: Ocular Trauma Score; NPL: No perception to light; PL: Perception to light; HM: Hand movement; N: Number

OTS	NPL		PL/HM		1/200 to 19/200		20/200 to 20/50		>20/40		N
1	21	55%	11	30%	6	15%	0	0%	0	0%	38
2	2	12%	5	33%	1	6%	4	26%	4	26%	16
3	0	0%	3	11%	6	22%	15	55%	3	11%	27
4	0	0%	0	0%	1	5%	7	30%	15	65%	23
5	0	0%	0	0%	0	0%	2	25%	4	75%	6

## Discussion

Our study showed that adult males possess the highest risks for ocular trauma. This could be attributed to the relatively higher tendency for risk-taking behaviour and that males constitute a larger proportion of manual workers. The racial distribution of ocular trauma in our studies is in keeping with the racial distribution of the working class in Kuala Lumpur with the Malays being the largest workforce. This study also found that most of the ocular injuries that require emergency surgery were sustained while at work. This is similar to numerous studies that were done locally and abroad.

A study by Omar et al. was done recently in Malaysia. They examined medical records of patients who have sustained workplace eye injuries and are registered with Social Security Services (SOCSO). Their study showed that males are 14 times more likely to sustain workplace eye injuries compared with females. The average age for all patients was 35 ±10 years while the average age for male and female workers who sustained workplace eye injuries were 34 ± 10 and 38 ± 12 years [3].

However, migrant workers account for a significant portion of ocular injuries too. Most of these workers are from the manufacturing or construction industry and unfortunately, health and safety measures such as protective eye goggles are often neglected among these workers at their workplace.

Another study done by Ngo and Leo in Singapore also revealed that of 300 patients who presented with an industrial accident-related ocular trauma, 95.7% were non-residents and were on average 31 years old and 99.3% were males [4]. The lower percentage of foreigners in our review could be attributed to the lower percentage of non-residents in the industrial or construction industry compared with Singapore.

A study done by Voon et al. (2001) in Singapore found that in 870 patients who presented with ocular trauma, they were 4.2 times more likely to be male, 6.2 times more likely to be non-residents and 3.2 times more likely to be younger than 40 years old [5].

Woo and Sundar (2008) examined 133 patients and 139 eyes who had eye injuries at the National University Hospital, Singapore. Their average age of patients was 33.5 and 84.2% were men, and 46.6% were non-Singaporeans. A total of 56.4% of eye injuries were sustained while at work and common sources of eye injuries included grinding, welding, hammering, human-inflicted injuries and road traffic accidents [6].

Lai and Moussa did a retrospective study at the Hospital University of Malaysia and studied 64 eyes with intraocular foreign bodies (IOFBs). Their study found that 70% of cases were below 35 years old, predominantly male (95.3%) and sustained their injuries at work (86%) [7].

Zainal and Goh also studied 167 eyes from 159 patients with perforating injuries who were treated at the Hospital University of Malaysia. In his study, the majority of patients were less than 40 years old, predominantly male. Common causes of injuries include occupational injuries (35%), motor vehicular accidents (29%) and domestic accidents (23%) [8].

Another study by Hooi and Hooi done at Johor Bahru also found that in 156 eyes with open-globe injuries, 88.2% were male and the majority were within the economically active age range of 15 to 64 years old. Most of the injuries were work-related [9].

Mallika et al. did a study in Sarawak General Hospital and reviewed 257 eyes from 233 patients. The cases were predominantly between the age of 21-30 years old. Common causes of injuries are work-related (36.9%). The places of injuries usually occurred at home (34.3%) or at an industrial premise (31.8%) [10].

Soong et al. [11] studied 603 eyes from 546 patients and found that 88.1% of patients were male with a male-to-female ratio of 7.4:1. Malaysians constituted 75.5% of the patients, of which 24.5% were foreigners. The average age was 31.5 years and 43.6% of the cases were work-related. Common causes of eye trauma include the use of high-powered tools (30.8%), motor vehicular accidents (23.1%) and domestic accidents (17.7%).

In our study, domestic household accidents accounted for 21% of ocular trauma. The majority of patients were children and most of them sustained injury while playing. A study done at Sarawak General Hospital also found that the majority (65.8%) of patients who sustained ocular trauma were boys aged 6.1 ± 3.0 years old and the injuries usually occurred at home [12].

This is similar to another study done by Puodžiuvienė et al. in Lithuania where they found that in the paediatric age group, boys are more likely to have ocular injury compared to girls. They also found that most injuries occur at home or at school and are caused by sharp or blunt objects [13]. This is also similar to another study done in the Philippines where it is found that young boys have a higher chance of sustaining an ocular injury caused by accidents at home [14].

A similar study in China also revealed that out of 1125 children, boys (73.8%) are more likely to be injured. Their injuries were more likely to be caused by sharp objects (48.4%), blunt objects (18.6%) and fireworks (10.8%) [15]. Parents and caretakers should place emphasis on preventive measures to avoid accidents from happening in the first place. This is because compared to adults, visual morbidity caused by trauma can lead to further issues such as amblyopia in children which hinder their development and affect their lives in the future.

In our study, most of the patients who required emergency surgery for ocular trauma sustained open-globe injuries, followed by adnexal injuries with only a small percentage of closed-globe injuries. As this review is only about patients who had undergone emergency eye operation, it cannot be used to characterize all eye trauma as the majority of patients who had closed-globe injuries or minor eye trauma would not have required emergency eye surgery at the operation theatre and would have simply received treatment at a primary care facility or the emergency department without being seen by the ophthalmology team. Although this review is not population-based, the implications in terms of morbidity should not be ignored.

In our study, the OTS is fairly accurate in predicting the final visual acuity after surgery. Most of the patients with adnexal or closed-globe injuries had a high OTS and hence had a good visual outcome. A majority of patients who had open-globe injuries had poor final visual acuity. This is consistent with the findings from the OTS where variables such as initial visual acuity, globe rupture, endophthalmitis, perforating injury, retinal detachment and an RAPD will contribute to a poorer visual outcome [1].

A study done at Palermo University for ocular trauma found that initial visual acuity was found to be correlated with the final visual acuity. The majority of the final visual acuity in the OTS categories correlated to that of the OTS study in the majority of the cases (12 out of 14) [16].

Another study by Beshay in Australia included 205 open-globe injuries. A total of 80 globe ruptures, 71 penetrating eye injuries, 48 IOFBs and six perforating injuries were included. The study found that globe ruptures and perforating injuries had poorer visual outcomes. Injuries with IOFBs were also associated with worse final best-corrected visual acuity. The study concluded that the initial presenting visual acuity is a good predictor for final visual acuity for non-ophthalmologists [17]. The OTS score is also accurate in the paediatric age group. The final visual acuity was positively correlated with the initial visual acuity and the OTS in children in a study for paediatric ocular trauma [15].

Watanachai reviewed 162 eyes who underwent surgery for IOFB between January 2015 and December 2020. The majority of the patients in this study have visual improvements following surgery with about 60% of patients having a final vision of 6/60 or better. This study also found that the OTS is a reliable estimator of final visual outcome in IOFB-related open-globe injuries in the higher OTS categories [18].

A study from Hospital Sibu, Sarawak found that open-globe injuries (61.5%) showed worse or no improvement in visual acuity compared to closed-globe injuries (28.9%) after three months (P = 0.015) [19]. The study in Hospital Universiti Sains Malaysia (USM) showed that the visual outcome was found to be significantly associated with the initial visual acuity (P <0.005), the posterior extent of the wound (P<0.001), length of the wound (P<0.001), presence of hyphaemia (P<0.001) and presence of vitreous prolapse (P <0.005) [20].

In an analysis of 167 perforating eye injuries from Hospital Universiti Kebangsaan Malaysia (UKM), at six months of follow-up, only 45% of eyes had improved vision to 6/36 or better, while the remaining 55% of eyes were blind. The extent of injury has been identified as the only significant associated factor. Age, time interval and mechanism of injury were not significant factors [8]. In a study of 156 eyes with open-globe eye injuries treated in the Hospital Sultanah Aminah, Hooi and Hooi found that prognostic factors included initial visual acuity, RAPD, wound location, lens injury, endophthalmitis and retinal detachment [9]. In a study of perforating injuries with IOFB at University Hospital, Lai and Moussa found that the size of the IOFB, poor initial visual acuity, and the presence of complications such as cataract, iris damage and vitreous haemorrhage affected the outcomes. Corneal opacity and amblyopia were the most common cause of poor outcomes among 29 children with traumatic cataracts [7].

Lee et al. did a retrospective study over 11 years at a tertiary trauma centre on 529 closed-globe injuries and adnexal injuries. In their study, the presenting visual acuity was a good predictor of the final visual acuity [21].

A study by Thevi et al. [22] in Hospital Tengku Ampuan Rahimah Klang found that predictors of good visual outcome are well-presenting visual acuity, corneal laceration wound with a size of less than 5mm, a deep anterior chamber and simple lacerations. Factors that did not affect the outcome include age, gender, place of injury, object causing injury, hyphema presence, and intraocular bodies.

As this study is a retrospective study, there might be limitations such as selection bias. This can be overcome by considering a prospective study in future. Secondly, as the case identification was made through surgical records, only patients who had undergone emergency surgery were identified and included in this study. Hence, the results may not reflect the true distribution of injury types and may have a bias towards eye trauma, which is more serious and requires emergency surgical intervention in the operation theatre. Therefore, the findings of this study may not be an accurate representation of the general population. Again, this may be avoided by doing a prospective study.

## Conclusions

From this study, we have identified that young male adults are most at risk for sustaining ocular trauma while at the workplace and are more likely to sustain open-globe injury with lasting visual impairment. Our study also found that the OTS is a helpful and reliable tool to help healthcare practitioners predict the final visual acuity based on initial findings. It is important to implement proper occupational safety and health laws in workplaces. Employers and workers should be educated on the importance of proper eye protection as this would greatly reduce the occurrence of ocular injuries and in turn reduce visual morbidity.
